# Microbial contamination of diesel-biodiesel blends in storage tank; an analysis of colony morphology

**DOI:** 10.1016/j.heliyon.2022.e09264

**Published:** 2022-04-09

**Authors:** Leily Nurul Komariah, Susila Arita, Muhammad Rendana, Cindi Ramayanti, Ni Luh Suriani, Desi Erisna

**Affiliations:** a Chemical Engineering, Department Faculty of Engineering Universitas Sriwijaya, Palembang, South Sumatera, 30139, Indonesia; b Chemical Engineering, Department State Polytechnic of Sriwijaya, Palembang, South Sumatera, 30139, Indonesia; c Biology Study Program, Faculty of Mathematics, and Natural Sciences, Udayana University, Denpasar, Bali, 80232, Indonesia; d Energy Engineering Laboratory Universitas Sriwijaya, Indralaya, South Sumatera, 30662, Indonesia

**Keywords:** Biodiesel, Colony forming unit, Contamination, Microbial growth, Storage tank

## Abstract

Fuel contamination is a major issue that comes with the utilization of biodiesel. Microbial growth is one of the primary causes of contamination during fuel handling and storage. This work attempts to identify the types, shapes, and growth profiles of microorganisms on fuel samples. The morphology of microbial colonies is presented in order to analyze the potential of fuel contamination. The diesel, biodiesel, and blends are stored in stainless steel (SS) and glass tanks, where each is placed indoors and outdoors during the 90 days of storage time. The morphology of microbial colonies is observed through a microscope with a magnification of 1000× and the quantity is calculated by a digital colony counter. Microbial contamination in all samples is considered as high contamination where the Colony Forming Unit (CFU) is greater than 10^5^ L^−1^. Colony forms are far more assorted in blends than in pure diesel (B0) and neat biodiesel (B100). The transformation of microbial colonies accelerates after 60 days of storage time. The results reveal that the number of bacterial colonies that grow in B20 is higher and more varied, nevertheless, the contamination in B100 is significantly higher. This is indicated by a 1.5-fold rise in B20 acidity and a 2.5-fold increase in water content compared to the initial condition.

## 1 Introduction

Biodiesel continues to be the best alternative for fossil diesel. In Indonesia, the government's mandatory for higher blend biodiesel in diesel fuel is raising up to 40% in the next year. An increase in the percentage of biodiesel may potentially cause contamination problems that lead to bigger operational problems. High quality and strict fuel hygiene to avoid damage, operation failures, and shortening the lifetime of the devices are needed for modern diesel engine technology recently.

In general, biodiesel has low oxidation stability, high lubrication ability, and is very hygroscopic. Many studies reported that biodiesel is more prone than petroleum diesel due to contamination and degradation [1, 2, 3, 4, 5, 6]. This susceptibility of biodiesel is associated with the chemical composition of the biodiesel and varies considerably depending on the raw material used [6]. Biodiesel is degraded through water vapor absorption, auto-oxidation, and microbial attack [7, 8]. The increase of microorganism populations has exhibited the ability to degrade the fuel storage tank and cause corrosion [9, 10, 11, 12].

Microbes are commonly found in fuel storage tanks, transport systems, and fuel supply chains. Biofilm production is triggered by microbial growth in storage tanks and pipes, which can block filters and pipelines, as well as increase pump and injection system wear. Fuel contamination shortens the filter's life and can result in fuel starvation, engine problems, and possible damage to the fuel injection equipment [13]. It confirms that the biodegradation of hydrocarbons is an integral part of microbial life [14].

The microorganisms found in petrodiesel, biodiesel, and its blends may vary greatly. Most of the researchers mentioned that almost all variants of microorganisms, such as bacteria, fungus, and yeast, may be found in diesel fuel, and biodiesel as presented in Table 1.

**Table 1:** Typical microorganisms detected in diesel and biodiesel storage tanks.

Common Diesel fuels		Biodiesel-Diesel Blends
Bacteria	Sulfate reducing bacteria (SRB), *Flavobacterium, Acinetobacter*, and *Micrococcus*	*Actinetobacter, Bacillus sp., Clostridium sporogenes, Flavofacterium diffusum, Micrococcus sp., Pseudomonas sp., Pseudomonas aeruginosa, Serratia marcescens, Sarcina sp., Hydrogenomonas sp., Clostiridum sp., Gordonia sp.,* etc.
Yeasts	*Candida, Saccharomyces, Torula*, *Hansenula*	*Candida sp., Candida famata, Candida lypolytica, Candida silvícola, Candida tropicalis, Rhodotorula sp., Saccharomyces sp.,* etc.
Moulds/Fungus	*Hormoconis resinae, Cladosporium resinae, Aspergillus, Penicillium, Fusarium and Botrytis*	*Acremonium sp., Aspergillus sp., Aspergillus fumigatus, Cladosporium sp., Fusarium oxysporum, Penicillium sp., Penicillium citrinum, Penicillium funiculosm, Trichiderma sp., Paecilomyces sp,. Moniliella and Byssochlamys, Phyla sp., Pseudallescheria boydii., Hormoconis resinae, Fusarium sp., Aureobasidium pullulans, Moniliella wahieum, Byssochlamys nivea,* etc.

Source: [1, 2, 7, 10, 15, 16, 17, 18, 19, 20, 21, 22, 23].

Numerous researchers report the presence of complex microbial diversity in diesel fuel and biodiesel. Different species have different degradation mechanisms, thus providing different results [10, 24, 25, 26, 27, 28].

Microorganism diversity, growth rate, and pattern can be affected by fuel constituents such as carbon and energy sources, chain structure of the compounds, presence of sulfur, storage, and environmental conditions (temperature, humidity, etc.) ([6, 22]). The microorganisms can develop in fuel systems and grow faster in warm and hot conditions. The risks of diesel fuel contamination get higher as it located in high humidity regions [29, 30, 31].

Most of the existing studies are more likely to show that the degradation and risk of contamination of biodiesel due to microbial growth tend to be linear with the increasing amount of biodiesel in the blend [ [1, 2, 7, 15, 17, 32]. The general conclusion is not necessarily generally accepted because it has not considered the complexity of the growth patterns of various types of microbes that are actually very dynamic and changeable depending on the availability of nutrients, storage environmental conditions, and storage tank materials. From the microorganism perspective, this will also relate to energy metabolism, endospore-forming, oxygen requirement, motility, etc.

Prevention of the growth of microorganisms in fuel storage tanks is a major concern for the industrial and/or commercial sectors because of the associated problems that are caused by corrosion, filter plugging, and blockage in storage, fuel lines, and/or dispensing facilities. The colony morphology in this study may also perform as a simple way to cross the confirm dynamic of the microbial community and also to identify the potential predominant species which responsible for biodiesel degradation and/or contamination. Most researchers assume that bacteria are more commonly found in contaminated diesel oil, while fungi are the microorganism most responsible for the microbial attack on biodiesel. However, few studies comprehensively link changes in physical properties of biodiesel with microorganism colony morphology in various storage conditions. Therefore, we conduct a novel study, the morphology of microbial colonies link to the fuel degradation and potential corrosion of steel and glass tanks fill with B0, B20, and B100. The output this study will give some recommendations for the design and operation of diesel-biodiesel blends storage tanks and they will potentially be generated and useful to reduce the risk of technical failure due to the microbial contamination.

## 2 Methods

The biodiesel used in this study is palm-based, which is blended with the splash blending technique into petroleum diesel oil, forming B20. Petroleum diesel (B0), neat biodiesel (B100), and B20 are stored in each of the two cylindrical storage tanks with flat and bottom roofs, stainless steel, and glass materials. One of the tanks is stored indoors, the rest is located outside. The observation lasted for 90 days, where the samples are taken every 30 days. The oil sample is taken from the zone of 1/4 level of tank height from the bottom, which is suspected water-oil interface position. A microscope and a colony counter the used to observe the morphology of microbe colonies and quantify the microorganism. Oil samples were taken by suction using a vertical pipe. The sampling point is at the bottom zone, precisely at a quarter of the oil level from the bottom. The method of acid number measurement is according to ASTM D664 and water content using Coulometric Karl Fischer Titration according to ASTM D1796. The colony morphology is used to identify the presence of microorganisms. In this work, a Stuart Digital colony counter is set for microorganism quantification. Plate counting is used to estimate the number of cells present based on their ability to form colonies under specific nutrient medium, temperature, and time conditions. Total Colony counts are quantified by the extent of visible microorganism colonies developed on nutrient agar plates in incubation at 24–37 °C. Then it is identified as Colony Forming Unit (CFU), CFU/mL means colony-forming unit per mL sample. Concentrations of colony-forming units can be expressed using logarithmic notation, where the value shown is the base 10 logarithm of the concentration.

The number of microbial (CFU) per mL or gram of sample is calculated by dividing the number of colonies by the dilution factor using Eq. (1).

(1) $$\frac{CFU}{mL}=\frac{\sum C}{\left[\left(1xn_{1}\right)+\left(0.1xn_{2}\right)\right]xd}$$

where, C = total colony in every plate, n_1_ = volume in first plate, n_2_ = volume in the second plate, d = level of dilution, dilution factors = 10^5^.

The CFU/ml can be calculated also using Eq. (2).

(2) $$\frac{CFU}{mL}=\frac{number\;of\;colonies\;x\;dilution\;factors}{volume\;of\;culture\;plate}$$

The morphological study is carried out and incubated around the storage tanks at ambient temperature (at average 28 °C) and relative humidity (up to 85%).

Factors contributing to the value of uncertainty for quantification of microbial colonies may be varied and influenced by the factors involve and interrelate. They include; dilution (sampling and weighing), sample homogenization, colony counter readings, equipment calibration, analyst/human, resolution, stability, bias, drift, repeatability, and reproducibility. In this study, we use a combination uncertainty analysis approach, although not all factors consider influential are taken into account. Accordingly, it is a function of the uncertainties of the dilution factor, the density estimate, the equipment reading, and the uncertainty of calibration. We analyze parameter distribution and statistical approach according to the 95% confidence intervals of parameters at each Markov chain based on the previous studies [33, 34].

## 3 Results and discussion

### 3.1 Visual Appearances on fuel degradation

**Figure 1:**
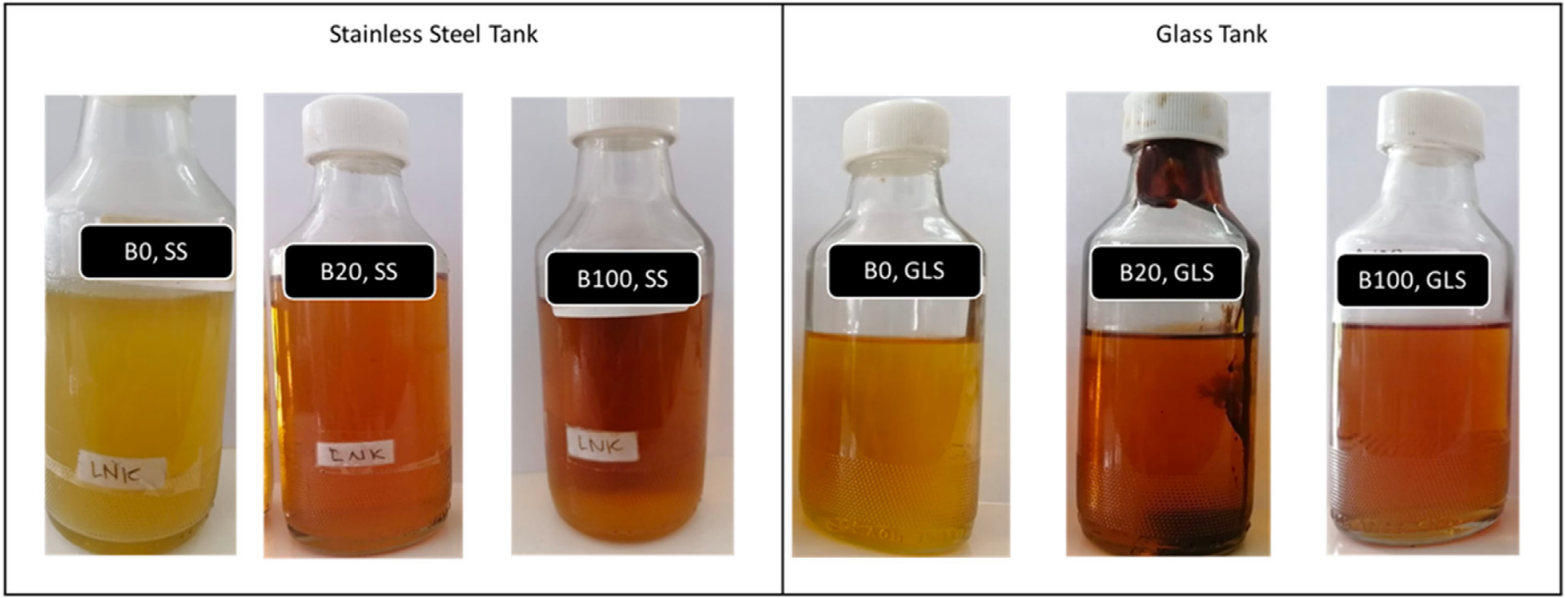
Visual Appearance for all fuel sample B0, B20 and B100 in SS tank and Glass tank after 90 days.

In this part, the degradation of diesel and biodiesel can be compared by the physical appearance of the fuel samples. After 90 days of storage, the detection of contamination in B0, B20, and B100 from the glass and stainless steel tanks have been presented, as shown in Figure 1.

Microbial contamination symptoms in diesel fuel might be seen as an aggregation of biomass, which showed up as discoloration, turbidity, and fouling. In this study, the process of forming biofilms, emulsions, and the presence of slime/sludge or deposits in all oil samples is observed microscopically. Discoloration or oil turbidity occurs in all samples, but surprisingly, the discoloration in B0 appears insignificant. Biofilms or biomass are not detected in the oil inside the B0 and B100 storage tanks, a small number of deposits are found at the bottom of the tank, but on the B20 stored in the glass tank, the presence of a thick, sticky sludge is clearly visible. As seen in Figure 4, it is clear that all stored oil has been contaminated at different levels. Turbid oil containing white floccose biomass is detected on samples of B20 and B100. In addition, a lump of brown biomass was seen in B20, which was accumulating especially more at the bottom glass tank.

Changes in the physical appearance of fuel samples indicate that degradation has occurred due to various causes and sources of contamination, particularly microorganisms. Fuel deterioration can result in turbidity, delamination, or deposit formation. In this study, significant turbidity on B20 and slime-shaped biomass are found after more than 90 days of storage. The color change has been detected since day 60th. The changes in the oil color and the presence of biomass prove the aggressiveness of the growth of microorganisms in the biodiesel-diesel blends. The biomass was found more in B20 than in biodiesel and diesel oil samples. As reported by Amaral et al. [30, 35] they found that B20 presented a viscous film at the end of the sixth month of aging, due to the presence of glycerides and the oxidative degradation products. Some microorganisms have the ability to break down the structure of the hydrocarbon in diesel [25]. However, this condition is particularly adverse to several authors [1, 2, 20, 36], which concluded that degradation occurs faster in B100 than in B0 and it blends.

### 3.2 Fuel degradation, microbial contamination, and changes in physical properties

**Figure 2:**
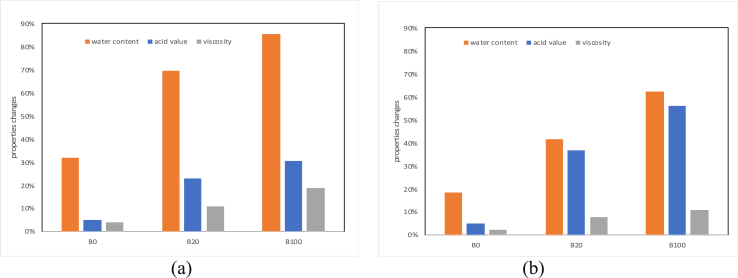
Changes in physical properties after 90 days of storage time. (a) Glass tanks, (b) SS tanks.

Microbial growth at high levels can change the qualities of fuel, especially after a long storage period. In this study, observations are more devoted to specific physical properties of oil fuel, such as water content, and acid number. They are considered to be strongly related to the level of fuel contamination, which is directly related to microbial growth. The most pronounced changes in properties occur in water content parameters and fuel acid numbers stored for more than 90 days. In this study, the highest change occurred in glass storage tanks placed outdoors as shown in Figure 2.

The most extreme change is to the sharply increased water content of B100 in both B100 and SS tanks, this is due to the elevated temperatures, local climate or the weather like sunshine-induced heating up of tanks. The contamination of B100 and B20 in glass tanks can be detected from the great changes in water content and acid levels. For B20 storage the increase in water content reached up to 153% from the initial condition, while the acid number elevated to 53%. While, on a sample of B100, it showed more extreme changes where the acid value increased 1.8 times and the water content increased 3.5 times from the initial condition. Fuel storage on SS tanks still shows changes in acid and water content even in lower levels than in glass tanks. This condition suggests that factors that allow high-air to enter through tank lid gaps and the increase in temperature outside cause the growth of microorganisms to become more dramatic.

During the storage period, it is known that the average temperature around the storage tank was 28 °C, and the air humidity was average at 88%. This condition is believed to accelerate the proliferation of microorganisms. Initial biodiesel samples are identified to have water content above quality standards due to the post-production storage conditions and transportation. The high dissolved water in the fuel, especially B20 will cause the formation of microdroplets, which are culminated or peak the productivity of the growth of microorganisms in free water found at the bottom of the tank. On glass and SS type tanks a layer of water and oil is visible at the bottom of the tank. The proliferation of microorganisms grows under such fundamental conditions. An increase in the water content of biodiesel may be due to inappropriate storage conditions, especially high temperatures. The presence of water leads to the hydrolysis of esters, resulting in the formation of free fatty acids and glycerol [2, 37, 38].

An increase in the water content in fuel is identical to an increase in its acidity. It is known that petroleum-based diesel fuel with high sulfur content triggers an increase in acid numbers, but in this study, the change during storage was not more than 11%. This condition indicates that the addition of 20% biodiesel to the fuel significantly triggers the growth and metabolism of microorganisms. Higher ambient temperatures also favour the growth of microorganisms.

#### 3.2.1 Microbial colony morphology

The properties of the colonies may help to assess the bacterium's identity. Bacterial colonies can be quite diverse depending on the species. The different colony forms on the type of fuel stored in metal and glass tanks indicate differences in the dominance of the microorganisms that grow in the oil. Table 2 shows the shape, edge, texture, consistency, color, size, and elevation of the colony formed in each fuel sample after 90 days of storage.

**Table 2:** Colony Identification, the general shape and chromogenesis

	colony identification/morphology-relative-dominant				
	colony shape	Edge	texture/consistency	color, size	elevation
B100	Irregular	irregular, lobate	irregular, wide floc	Non pigmented, Medium to large	flat, and raised
B20	Irregular, circular	irregular, lobate	scattered, adjacent	Non pigmented, small	flat
B0	Circular	irregular, lobate	scattered, adjacent	Non pigmented, Punctiform, small	flat

The microbial colonies on B0, B100, and B20 had different appearances, shapes, and distribution patterns. The shape of bacterial colonies in B100 was relatively more prominent, and the distribution pattern was reasonably consistent, both found in glass and SS tanks.

**Figure 3:**
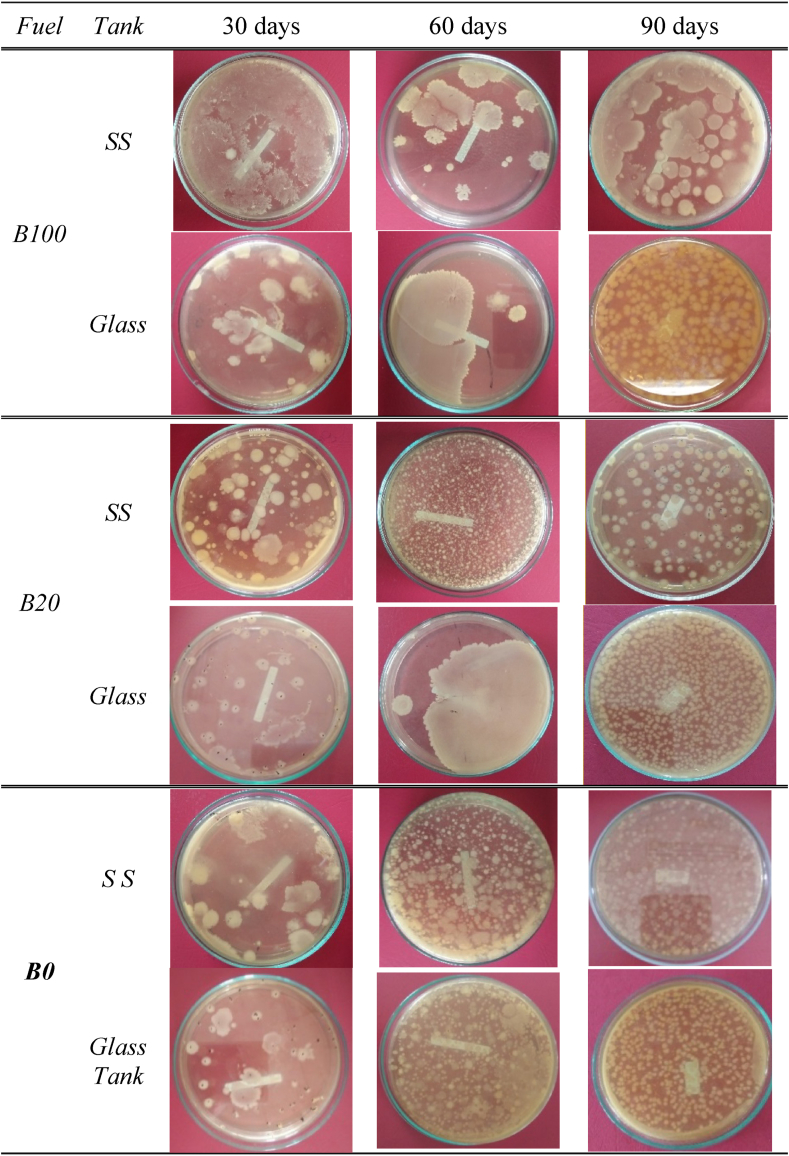
Microbial colony profile for B0, B20 and B100 in storage tanks.

As seen in Figure 3, colonies compose a wide distribution with smaller sizes in storage conditions exposed to the environment (glass tank). In contrast, the shape of bacterial colonies on B0 and B20 was dominated by the small settlements. Thus, it tends to spread close together and relatively even. This condition indicates the type and transformation of the type of microorganism that grows in it.

In general, colonies formed on sample B20 on day 90 of storage tended to be similar to the colonies settlements on B0. At the same time, the transformation of colony growth on B20 tended to be random. B20 stored in the first 30 days showed similar colony formation patterns in both samples stored in SS tanks and glass tanks. However, after more than 30 days of storage, in B20, which was stored exposed to light, a phase occurred where the colonies seemed to combine to form flocs and then spread into small colonies with an even distribution.

### 3.3 Quantity of microbial colony

In most CFU estimates, each colony is assumed to be distinct and generated by a single live microbial cell, as presented in Table 3. Therefore, the concentration of colony-forming units can be expressed by logarithmic notation, where the value displayed is logarithmic base 10 (Log CFU).

**Table 3:** Number of Bacterial Colonies in fuel oil samples after 90 days of storage.

Storage condition	Tank	Colony Forming Unit (CFU/mL) in fuel sample		
		B0	B20	B100
Indoor	SS	2,60,E+07	3,40,E+07	6,60,E+07
	Glass	6,20,E+07	1,13,E+08	1,17,E+08
Outdoor	SS	8,60,E+07	1,24,E+08	1,06,E+08
	Glass	6,63,E+07	1,45,E+08	1,08,E+08

**Figure 4:**
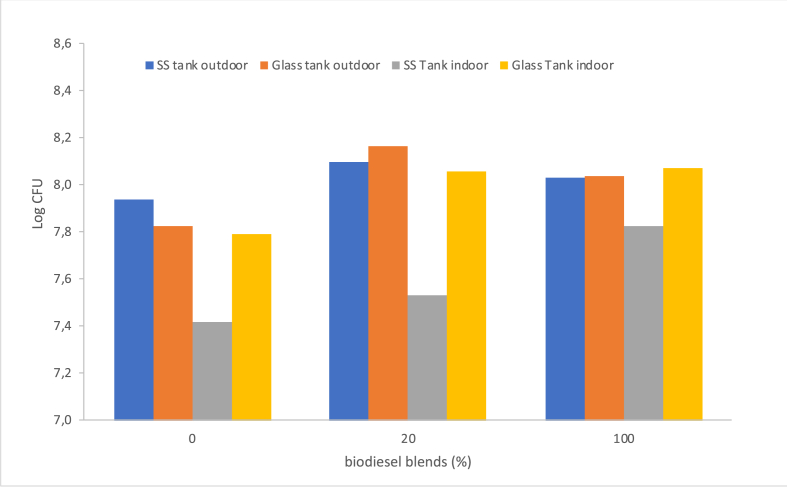
Logarithm of colony forming units in B0, B20, and B100 in SS and Glass Tanks.

The number of bacteria detected on the sample plate on the Colony Counter of each oil sample increased with the length of storage time. In general, the number of microbial colonies in oil samples stored in glass tanks was more than those stored in SS tanks. As seen in Figure 4, the number of colonies formed in B20 is equivalent to that of B100 and/or tends to be higher.

The measurement results in this section contain uncertainty that comes from various sources. Referring to several studies, the most significant uncertainty sources for derived biological quantities are those related to the assigned value of the end-user calibrator, the long-term intermediate precision, and the bias [39, 40, 41]. The calibration error depends on the accuracy of the calibration standard. Calibration Error from the equipment manufacturer of the colony counter can be estimated by error limit divided by 2 [33]. The colony counter calibration reference standard used refers to manufacturer data (Stuart Colony Counter) which is also confirmed and reported by [42, 43, 44]. The test results show that the uncertainty factor for reading Colony Counter digital data is 0.1 log CFU and the percent calibration uncertainty is in the range of 1.19–6.8%.

In order to simplify the mathematical expression, we have only considered the whole dilution series as one process that results in the dilution factor, F. A typical result with dilution steps 1:10 and higher, presenting dilution factor f = 10^6^, the standard uncertainty w_f_ = 0.02 (2 %). In colony counter operation, the average ratio between found colonies and actual colonies on an image was 0.75 with a deviation standard of = 0.26 indicating a general underestimation of colony numbers. From dilution calibration experiments, it is found that the relative standard uncertainty of the 1 mL inocula is w = 2 % and that of the 0.1 ml measurements equal to w = 8 % as also reported by [43].

The number of microbes that grow in SS tanks is lower than in glass tanks, both indoor and outdoor. The increase of microbial quantity is linear with biodiesel composition in fuel only applying to samples stored in the SS tank. The highest microbial growth occurred in the B20 sample. The quantity is relatively higher than microbial detected in B100 and B0 at all storage conditions. B20 appears to be more prone to degradation than B100. This condition is related to the extreme changes in physical properties that occur during storage. Horel and Schiwer [22] explained the most easily degradable hydrocarbon fuel components are medium-chain compounds that can promote the rapid growth of local microbial communities. Biodiesel blends (B20) are still 80% dominated by petroleum oil so it contains straight-chain alkanes (paraffin). This molecular bond tends to be more readily degraded by microorganisms than aromatic and alkenes (olefins). As described by Groysman [45], microorganisms do not grow much in fuel with more olefins and aromatics, it is due to microbes consuming hydrocarbons of higher molecular weight available in fuel. However, the presence of aromatics and oxygenates in fuels, as well as the addition of biodiesel in the blends, cause an increase in water solubility in fuels.

Colonies of microorganisms that grow on petroleum diesel oil generally come from groups of fungi and bacteria. The presence of a mixture of 20% biodiesel is very likely to cause the growth of certain species to stop but trigger the growth of other species in the same genus and or different with different growth patterns. This condition is related to the nutrients provided and the 20% reduction in sulfur content in the oil. The possible microorganisms that survive and grow become more diverse with the length of storage time.

As stated by Martin-Sanchez et al. [7], the diversity of microorganisms contained in the mixture of biodiesel blends is considered low, but has a longer survival ability, due to the availability of a number of complete nutrients and energy. There was a slight change from bacterial growth to fungal growth with increasing biodiesel in fuel blends.

**Figure 5:**
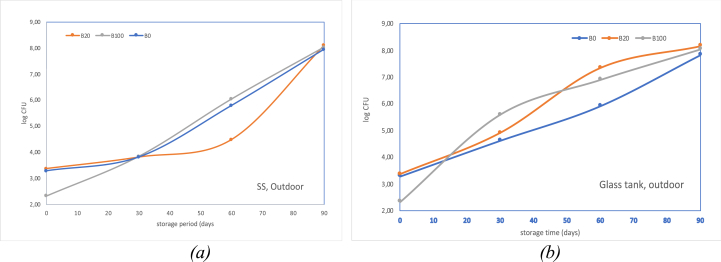
Profile colony growth in B0, B20, and B100 in (a) SS tank (b) glass tank (outdoor).

From the comparison seen in Figure 5, the increase of bacterial colonies in two types of storage tanks, both tended to show a progressive increase. It shows a dramatic increase after 60 days of storage. Micro-organism proliferation in B20 samples showed more volatile growth patterns than B0 and B100 that were relatively constant increases. In an outdoor glass tank, the highest microbial populations were seen on B20.

The quantity of CFU produced in B100 is on average 56% higher than the starting condition, compared to 34% and 36% respectively in B0 and B20. This term is correlated with the variety of nutrients and surplus carbon sources are provided by microorganism growing media, as well as the lower sulfur level in the fuel mixture due to the increased use of biodiesel in blends. The results of our study are also in line with a study by Manuel Restrepo-Florez et al. [46] they also assumed that the number of microbes that grow on B100 is relatively less than B20 and B0. Microbial counts present a sharp increase during the first month of storage due to an excess of carbon sources. At higher biodiesel concentrations, a statistically significant decrease in microbial counts is observed. A statistical reduction in microbial counts is also found in pure biodiesel samples (B100) after 50 days of storage. While the microbial activity is detected by Mitter et al [47] in the first 5 weeks of fuel contamination.

Based on this study, it is obtained that the quantity of biodiesel in the blends is not constantly linear with the rate of fuel degradation due to microbial attacks. These findings are very different from other previous studies. But all the fuel samples, with and without biodiesel, are particularly susceptible to microbial attacks in a storage period of 2 months or more. Colony dynamics that are shown from the morphological analysis proves microorganisms that survive on fuel samples in storage conditions support their growth with energy and the availability of nutrients.

## 4 Conclusion

The morphology of the colonies and fuel property change indicate changes in the atmosphere of microbial growing media for a living. The blending of B0 and B100 causes the oscillation of microbial colony growth, also exacerbate the level and rate of fuel deterioration. The level of microbial contamination that occurs in all fuel samples is categorized as high-contamination (higher than 10^5^ L-1). The morphology colony analysis shows a potential change in the kind of microorganism (bacteria to fungal) with increasing biodiesel in fuel blends (B20). At storage or incubation time of more than 60 days, the number of microorganisms changes and grows dramatically, especially in B20 stored in glass tanks. The microbial contamination rate in B20 appeared to be faster than others, but the worst fuel deterioration occurred in B100, especially in glass tanks placed outdoor. Fuel stored in storage tanks has abundant nutrients and enough energy for microorganisms to grow more at risk of fuel contamination and degradation. These findings will significantly affect fuel storage and handling strategies, including the design of the storage tanks.

## Declarations

### Author contribution statement

Leily Nurul Komariah: Conceived and designed the experiments; Performed the experiments; Analyzed and interpreted the data; Contributed reagents, materials, analysis tools or data; Wrote the paper.

Susila Arita: Conceived and designed the experiments; Contributed reagents, materials, analysis tools or data.

Muhammad Rendana: Analyzed and interpreted the data; Wrote the paper.

Cindi Ramayanti: Performed the experiments; Contributed reagents, materials, analysis tools or data.

Ni Luh Suriani: Analyzed and interpreted the data.

Desi Erisna: Performed the experiments.

### Funding statement

This research did not receive any specific grant from funding agencies in the public, commercial, or not-for-profit sectors.

### Data availability statement

Data included in article/supplementary material/referenced in article.

### Declaration of interests statement

The authors declare no conflict of interest.

### Additional information

No additional information is available for this paper.
